# Reply to the Commentary on population matched (pm) germline allelic variants of immunoglobulin (*IG*) loci: relevance in infectious diseases and vaccination studies in human populations

**DOI:** 10.1038/s41435-021-00155-3

**Published:** 2021-12-07

**Authors:** Indu Khatri, Magdalena A. Berkowska, Erik B. van den Akker, Cristina Teodosio, Marcel J. T. Reinders, Jacques J. M. van Dongen

**Affiliations:** 1 Department of Immunology, Leiden University Medical Center, 2333 ZA Leiden, The Netherlands; 2 Leiden Computational Biology Center, Leiden University Medical Center, 2333 ZC Leiden, The Netherlands; 3 Department of Molecular Epidemiology, Leiden University Medical Center, 2333 ZC Leiden, The Netherlands; 4 Delft Bioinformatics Lab, Delft University of Technology, 2628 CD Delft, The Netherlands

**Keywords:** Immunogenetics, Haplotypes

**Dear editor,**

We previously published our pmIG database, where we profiled the population matched (pm) germline alleles for the immunoglobulin (*IG*) locus [1]. To obtain the germline alleles, we simply used variant calling format files from the 1000 Genomes resource comprising 2504 individuals from 26 populations distributed within 5 different ethnicities, i.e., Africans, Americans, East Asians, Europeans, and South Asians. The major aim of the pmIG database was to understand the diversity of the germline *IG* alleles, i.e., unique or shared in different populations. To reach our aim, along with profiling the germline alleles, we also made the haplotype frequency for each allele available to our users. Such information is missing from the current germline resources, i.e., IMGT, IgPdb, VBASE2, and OGRDB. Furthermore, we also divided our alleles into the category of the previously known (AS1: high confident), novel but frequent (AS2, supported by >19 haplotypes), and novel and rare (AS3, supported by 7–19 haplotypes) alleles. This division allowed us to estimate the known and novel alleles from the 1000 Genomes resource.

**Fig. 1:**
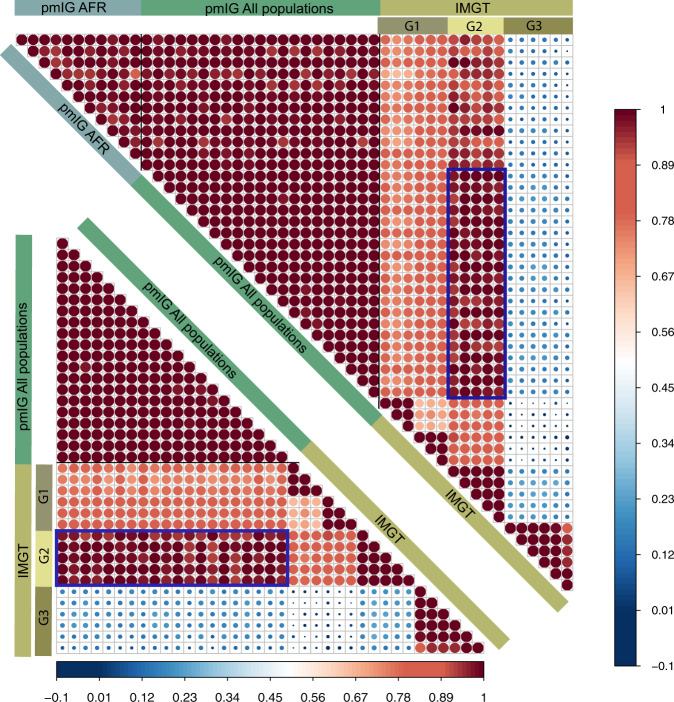
Correlogram representing the similarity of the IMGT artificial loci with the pmIG artificial loci belonging to all populations. To develop the artificial loci, we randomly selected the alleles for *IGHV1* genes and concatenated the alleles in a particular order of the *IGHV1* genes (*IGHV1-18*, *1-2*, *1-24*, *1-3*, *1-45*, *1-46*, *1-58*, *1-69*, and *1-8*). For pmIG alleles, we separated the alleles specific to the African populations (represented by “pmIG AFR”) and the alleles present in all the populations (represented as “pmIG All population”) and generated the artificial loci with respective alleles. These artificial loci were aligned and the aligned regions containing gaps and the unknown nucleotides (“N” bases in the IMGT alleles) were discarded. The selected aligned regions were converted into a binary form of the matrix and Pearson correlations were computed. The upper correlogram comprises of all the three categories of the artificial loci, i.e., “IMGT,” “pmIG All populations,” and “pmIG AFR,” IMGT loci in G1 category showed ~70% correlation with other IMGT alleles and all pmIG alleles. Interesting, G2 category of the IMGT loci shows a higher correlation with the pmIG loci from all populations (>90%) as compared to the ones derived from African alleles (~80%), as outlined by the box in blue. In contrast, G3 category of the IMGT artificial loci did not show any similarity to any of the IMGT or the pmIG alleles. This category of the alleles is comprised of the heavily mutated *IGHV1-69* alleles from the IMGT database. The lower correlogram is a subset of the upper correlogram that does not comprise of the African-specific pmIG artificial loci. We can clearly observe the high similarity of the G2 category IMGT loci with the pmIG loci derived from the alleles belonging to all the populations (majorly consisting of the GRCh37 genes of which IMGT is also majorly comprised of). This overall data again support our claim for the lack of population-based diversity in the IMGT database (and other existing databases).

We are aware of the complex endeavors required to develop a database for the *IG* germline alleles. The complexity is majorly because of the vast duplication levels present in the genes of the *IG* loci (in contrast to the T-cell receptor genes). In the past two decades, the state-of-the-art IMGT database has put great efforts into compiling such a resource. Our efforts to build the pmIG database were never meant to replace the IMGT database, but “to be used together with the IMGT database” to better understand immune repertoire studies and vaccination studies in different ethnic populations. We believe that the pmIG database holds two major advantages as compared to the other databases, detailed as follows:

1. **The immune repertoires in different populations can be driven by their respective germline allele makeup**. Henceforth, Caucasian germline alleles should be used to assess the immune response repertoire generated from Caucasian populations. Using the Caucasian germline pool for assessing African immune response repertoires or vice versa will lead to a biased assessment of mutation processes. All available databases, i.e., IMGT, IgPdb, VBASE2, and OGRDB, except the pmIG database, are developed majorly on alleles obtained from Caucasian individuals. The high similarity of the IMGT alleles to the pmIG alleles present in all the populations as compared to the African alleles (Fig. 1) supports the lack of African-specific alleles in IMGT. This lack of population diversity in the IMGT and other databases was the major reason we set out to understand the difference in the populations at the germline level in *IG* loci. Moreover, understanding the diversity of the African (Fig. 2A) and East-Asian (Fig. 2B) super-populations in the 1000 Genomes database suggests that the existing databases might lack the alleles associated with the East-Asian super-populations as well. Interestingly, we do not observe a high variability among African populations (Fig. 2C), suggesting a common ancestry for the African individuals sampled for the 1000 genomes.

**Fig. 2:**
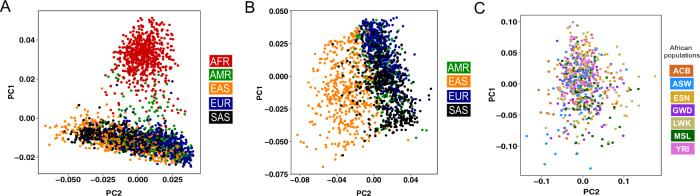
High diversity in African and East-Asian populations in the *IG* loci from 1000 Genomes resource. The PCA plots are generated using the SNPs in the *IGH* loci in the 2504 individuals available in the 1000 genomes. Each individual is colored based on the (super)populations these individuals belong to. **A** PCA plot using all individuals in the 1000 Genomes that clearly suggest that African (AFR) individuals have a higher diversity and are genetically distinct from other super-populations. **B** PCA plot generated by excluding the African individuals to understand the diversity among other populations. After excluding the African super-populations, a clear separation of East-Asian (EAS) super-populations is observed as compared to the America (AMR), European (EUR), and South-Asian (SAS) super-populations. **C** PCA plot generated using only the African populations wherein we observe a homogenous mixing of the African populations, suggesting a common ancestry of the populations sampled in the 1000 Genomes. Please note that the majority of these populations are sampled from the western coast of the Africa. ACB: African Caribbean in Barbados; ASW: African Ancestry in Southwest US; ESN: Esan in Nigeria; GWD: Gambian in Western Division, The Gambia – Mandinka; LWK: Luhya in Webuye, Kenya; MSL: Mende in Sierra Leone; and YRI: Yoruba in Ibadan, Nigeria.
2. **The pmIG database provides the estimated frequency of a particular allele in different populations**. Given the fact that a polymorphism can be specifically involved in infectious disease or vaccination studies in human populations, users of the pmIG database can now search for such correlations. Examples of such observations were elaborated in our publication, where we presented the frequency of particular alleles of *IGKC*, *IGHV1-69*, *IGHV3-23*, and *IGHV4-61*, which were associated with *Helicobacter pylori* infection in gastric cancer and age in breast cancer, Influenza responses, *Haemophilus influenza* type b and higher risk of rheumatic heart disease, respectively.

The two above-mentioned features are unique to the pmIG database as none of the currently available databases comprise such detailed information. Neither the source nor the frequency of the various *IG* gene alleles is clearly outlined in other databases. It is generally overlooked that the vast majority of each individual *IG* gene and allelic variant in other *IG* databases are in fact single genes, each derived from a single individual. Also, we believe that the conservative approaches used by these resources cannot reach such a detailed level of information for the germline alleles. To revolutionize the population-specific understanding of infectious diseases and vaccination responses, we should use the vast amount of the genome sequencing datasets, available in the public domain.

Nevertheless, like others in the field [2], we fully realize the shortcomings of the short-read-derived genome mappings. Therefore, to guide the users, we consistently mention the limitations of our database, which we also clearly emphasized in the discussion of our original manuscript. In line with this awareness, we herewith explain our current progress and actions regarding the developments associated with the pmIG database.

1. **A continuous process of updating the pmIG database according to publicly available datasets including long-read sequencing dataset**: We developed our resource using an older version of the human genome, i.e., GRCh37 mapping from the 1000 Genomes database. Therefore, sequences of a few genes are updated in the latest version of the human genome, i.e., GRCh38. For example, an additional base present in GRCh37 version of *IGHV3-49* and *IGHV3-53* reference genes is now removed in the GRCh38 version of the human genome. Such differences certainly will impact the alleles for these genes in the pmIG database. Therefore, we are currently working to identify such gene disparities in all the *IG* genes to update our pmTRIG database (https://pmtrig.lumc.nl/) accordingly. We will further publish the updates to keep our users up-to-date. Moreover, now that the long-read dataset is made available by the 1000 genomes, we will most likely be able to resolve several issues related to the short reads.
2. **Monitoring of erroneous pmIG alleles with special attention to AS3 alleles**: As already discussed in our manuscript, we herewith emphasize again that the pmIG dataset was derived from short-read sequencing data and that consequently mapping errors in the duplicated genes may exist, such as the mapping errors mentioned by Collins et al. for genes *IGHV3-11* and *IGHV3-48* [3]. We have highlighted genes with similar possible errors in Supplementary Table 5 in our original manuscript [4]. Especially alleles of the AS3 category might be problematic and should be used carefully for the repertoire assessment. Therefore, we are documenting these AS3 alleles on our website.

Furthermore, we would like to emphasize some facts of the pmIG database that are different from the IMGT database. Following unique facts about the pmIG database should be considered by our users before using the pmIG database for repertoire assessment in population-specific infection and vaccination settings:

1. **The pmIG gene and allele sequences are complete**: We have obtained complete gene sequences from the human genome using GENCODE gene annotations. Consequently, we would like to emphasize that the gene (henceforth allele) sequences in the pmIG database are complete and no truncation in the 5’ or 3’ end is present. One might observe additional bases in the IMGT genes (e.g., “ga”), but these bases neither code for any amino acid nor they are assigned any numbering by the IMGT database.
2. **pmIG allele identifiers are unique**: The pmIG database assigns gene names to all the alleles, even if the polymorphisms exist in the leader region of the *V* gene. Such identifiers allow us to keep the frequency of the alleles intact for any known polymorphism in the complete *V* gene. To facilitate the use of our database in immune repertoire studies, we have provided the gapped *V* exons per category (AS1–AS3), as per IMGT gapping and numbering. In addition, we have provided the gapped alleles per population category for population-specific repertoire studies.
3. **Use European population-specific pmIG alleles in comparison with IMGT database**: In line with our previous point, we would like to urge our users to use the Caucasian-specific germline alleles for Caucasian population-specific repertoire analysis. We mentioned that the majority of the novel alleles that had additional polymorphisms are majorly African populations specific. Owing to differences in population-origin of the IMGT and pmIG database, the mutation patterns will differ if users use one or the other resource for repertoire studies.
4. **Using combined IMGT and pmIG alleles for repertoire assessment after cleanup**: We also consider providing our users with the alleles with an identifier similar to the IMGT alleles so that the users can use a combined resource for assessing repertoire. This will allow users to add the genes missing in the pmIG database which in return will be helpful to avoid wrong germline calls for the repertoire. For the pmIG database, we will also provide the alleles that can be validated using Sanger/long-read sequencing by independent research groups [5]. Furthermore, one should consider assessing the possible false-positive alleles in the IMGT database [6] when used for repertoire studies.

Overall, we know the limitations and pitfalls of using short reads to build a germline database. Therefore, we are continuously finding ways to address such issues instead of just not considering the alleles such as in case of duplicated genes. For now, we propose using our data as supporting information to the IMGT alleles and comparative purposes whenever novel alleles are identified in your datasets. This will allow us to keep updating our resource with the information on the novel alleles. Henceforth in future, the pmIG database can be a complete database with complete information regarding the confidence of the alleles, population specificity, and frequency of alleles in different human populations. Furthermore, we aim to extend our database with alleles from populations that we could not gather from the 1000 Genomes database. In doing so, we aim to have a significant impact on studies related to population-specific immune responses in health and disease.
